# XAF1 forms a positive feedback loop with IRF-1 to drive apoptotic stress response and suppress tumorigenesis

**DOI:** 10.1038/s41419-018-0867-4

**Published:** 2018-07-24

**Authors:** Seong-In Jeong, Jung-Wook Kim, Kyung-Phil Ko, Byung-Kyu Ryu, Min-Goo Lee, Hyo-Jong Kim, Sung-Gil Chi

**Affiliations:** 1 Department of Life Sciences, Korea University, Seoul, 02841 Korea; 2 Department of Internal Medicine, Kyung Hee University Hospital, Seoul, 02447 Korea

## Abstract

X-linked inhibitor of apoptosis (XIAP)-associated factor 1 (XAF1) is a proapoptotic tumor suppressor that is frequently inactivated in multiple human cancers. However, the molecular basis for the XAF1-mediated growth inhibition remains largely undefined. Here, we report that XAF1 forms a positive feedback loop with interferon regulatory factor-1 (IRF-1) and functions as a transcriptional coactivator of IRF-1 to suppress tumorigenesis. Under various stressful conditions, *XAF1* transcription is activated by IRF-1, and elevated XAF1 stabilizes and activates IRF-1. Mechanistically, XAF1 binds to the multifunctional domain 2 of IRF-1 via the zinc finger domain 6, thereby hindering C-terminus of Hsc70-interacting protein (CHIP) interaction with and ubiquitination of IRF-1. Activation of the IRF-1−XAF1 loop greatly increases stress-induced apoptosis and decreases the invasive capability of tumor cells. Oncogenic Ras and growth factors interfere with the IRF-1−XAF1 interplay via Erk-mediated repression of *XAF1* transcription. Furthermore, XAF1 enhances IRF-1-mediated transcription of proapoptotic genes via the XAF1-IRF-1 complex formation on these target promoters. Meanwhile, XAF1 inhibits NF-κB-mediated tumor cell malignancy by reinforcing IRF-1 binding to a subset of coregulated promoters. Expression levels of IRF-1 and XAF1 correlate tightly in both cancer cell lines and primary tumors, and XAF1-induced tumor regression is markedly attenuated in IRF-1-depleted tumors. Collectively, this study identifies a novel mechanism of XAF1-mediated tumor suppression, uncovering XAF1 as a feedback coactivator of IRF-1 under stressful conditions.

## Introduction

X-linked inhibitor of apoptosis (XIAP)-associated factor 1 (XAF1) is a proapoptotic tumor suppressor whose expression is commonly inactivated in a broad range of human malignancies mainly by aberrant promoter hypermethyaltion^1–5^. Epigenetic silencing of *XAF1* is associated with the stage and grade of many tumors, supporting the implication of its inactivation in the malignant progression of tumors^3–5^. A recent integrated analysis of data from a PTEN loss-driven mouse model and cancer patients demonstrated that XAF1 downregulation is a predictive and actionable signature of castration-resistant prostate cancer^6^. The *XAF1* gene encodes 33 kDa protein that contains seven tumor necrosis factor (TNF) receptor-associated factor (TRAF)-like zinc finger (ZF) domains, suggesting its role in the regulation of protein−protein interaction^1,2^. Several isoforms of *XAF1*, including the full-length transcript (*XAF1A*, hereafter referred to as *XAF1*) and short truncated transcripts (*XAF1B-E*), are expressed in normal tissues^5,7^. Among these isoforms, *XAF1A* is preferentially lost or downregulated in tumors whereas truncated isoforms, such as *XAF1C*, are rather upregulated^5,8^. Our recent study showed that compared to XAF1A, XAF1C has considerably low apoptosis-promoting and growth-inhibiting activities both in vitro and in vivo, supporting that preferential loss of *XAF1A* and concurrent isoform switch contribute to tumor progression^9^.

XAF1 was first identified as a nuclear protein that binds and antagonizes the anticaspase activity of XIAP by inducing the nuclear translocation of cytoplasmic XIAP^1^. It was thus proposed that altered expression of XAF1 may elevate the cytoplasmic XIAP protein level, thereby deregulating the apoptotic caspase signaling^2^. However, the nuclear translocation of XIAP is not recognized in certain tumor cells undergoing XAF1-driven apoptosis and that XAF1 has comparable proapoptotic activity in *XIAP*^−/−^ and *XIAP*^+/+^ cells, indicating that XAF1 can promote apoptosis through the XIAP-independent mechanisms^5,10^. A growing body of evidence demonstrate that XAF1 activates caspases and increases tumor cell response to various apoptotic stresses, including γ-irradiation, fluorouracil (5-FU), H_2_O_2_, and growth factor withdrawal, and regulates autophagic cell death, tumor angiogenesis, and G2/M checkpoint of the cell cycle^11–13^. Our study has shown that XAF1 is a feedback activator of the p53 tumor suppressor and functions as a molecular switch in p53-mediated cell-fate decisions favoring apoptosis over cell-cycle arrest^9^. In this process, XAF1 appears to bind to p53 and interferes with mouse double minute 2 (MDM2) binding and ubiquitination of p53, and promote homeodomain-interacting protein kinase 2 (HIPK2)-mediated p53 phosphorylation and zinc finger protein 313 (ZNF313)-mediated p21^WAF1^ ubiquitination. Recently, we also reported that XAF1 is induced by heavy metals and triggers an apoptotic switch of stress response by binding and destabilizing metallothionein 2A (MT2A)^14^. XAF1 is an interferon (IFN)-stimulated gene that enhances IFN-induced apoptosis and strongly influences IFN-mediated cellular sensitization to the proapoptotic actions of TNF-related apoptosis-inducing ligand (TRAIL)^15–17^.

Interferon regulatory factor (IRF)-1 is the first member of the IRF family of transcription factor that is originally identified as a key regulator of type I IFN (α/β)^18,19^. IRF-1 is induced by viruses, lipopolysaccharide (LPS), interleukin-1 (IL-1), IFNs, and TNF, and plays a critical role in the regulation of host defense, such as innate and adaptive immune responses, viral infection, inflammation, and autoimmunity^20^. IRF-1 also acts as a tumor suppressor by modulating expression of genes involved in apoptosis, cell-cycle control, and angiogenesis^21–24^. IRF-1 is expressed at low levels by most types of resting cells but transcriptionally upregulated by the Janus kinase (JAK)-signal transducer and activator of transcription (STAT) or NF-κB pathway in viral infected cells or by the ataxia-telangiectasia mutated (ATM) pathway in response to genotoxic stress^18,25^. IRF-1 is inactivated by genetic and epigenetic mechanisms in multiple human malignancies, including myeloid leukemia^26–28^. IRF-1 protein turns over rapidly with a short half-life and several ubiquitin E3 ligases, including C-terminus of Hsc70-interacting protein (CHIP), have been shown to play a key in the regulation of IRF-1 stability^29,30^. However, the molecular mechanism by which IRF-1 is controlled to evoke appropriate responses to various stimuli remains to be characterized.

In the present study, we demonstrate that XAF1 forms a feedback loop with IRF-1 under stressful conditions and evokes its tumor suppression effect in a highly IRF-1-dependent fashion. Our data illuminate the potential mechanistic consequences of the IRF-1−XAF1 loop disruption in human cancers.

## Results

### IRF-1 and XAF1 cooperate to promote apoptosis under various stressful conditions

**Fig. 1:**
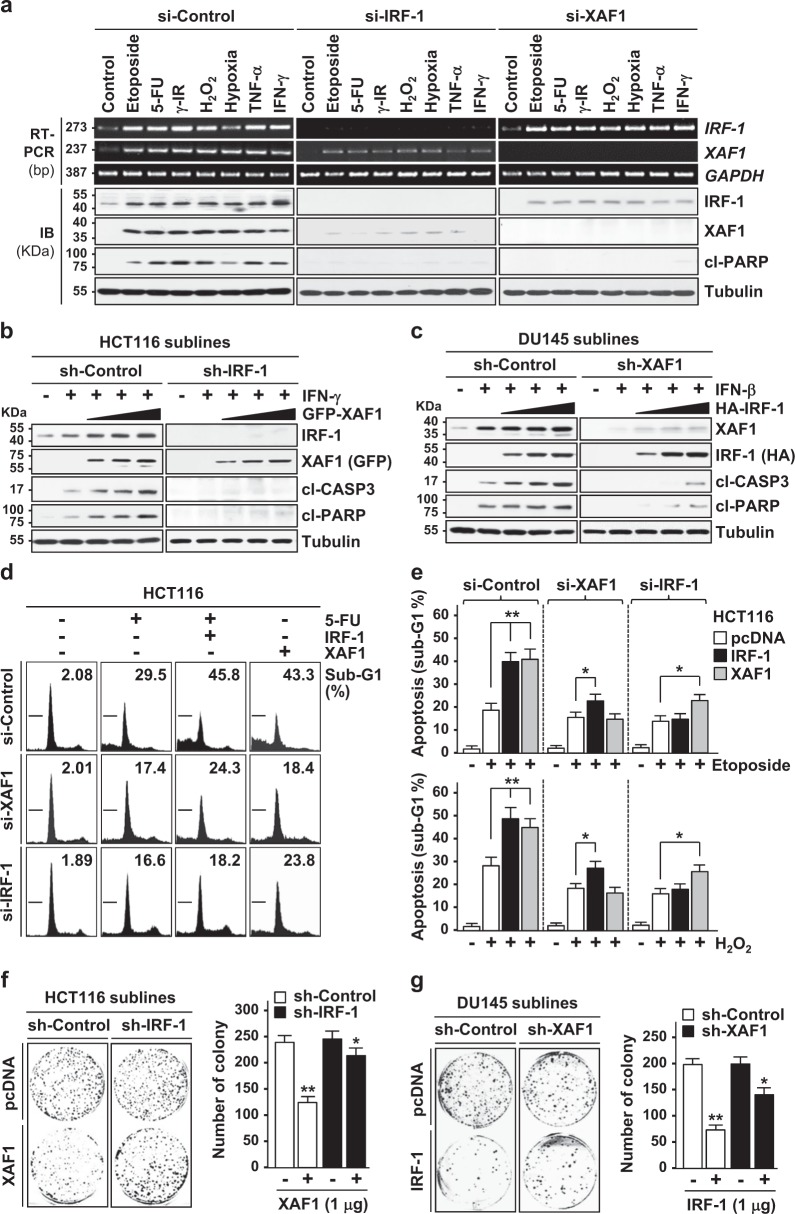
An XAF1-IRF-1 interrelationship in stress-induced apoptosis. **a** Induction of IRF-1 and XAF1 by various stresses and their relationship in the regulation of stress-induced apoptosis. HCT116 cells were transfected with siRNA (20 pM) as indicated and exposed to etoposide (50 μM), 5-FU (25 μM), γ-IR (6 Gy), H_2_O_2_ (50 μM), hypoxic condition (1% O_2_), TNF-α (50 ng/ml), or IFN-γ (0.5 μg/ml). After 48 h exposure, immunoblot (IB) and RT-PCR (reverse transcription-PCR) assays were performed. cl cleaved. **b**, **c** The mutual dependency of XAF1 and IRF-1 in apoptosis induction. Short hairpin (sh) RNA-expressing stable cells were transfected with empty vectors (pcDNA3.1) or increasing doses of green fluorescence protein (GFP)-tagged XAF1 or hemagglutinin (HA)-tagged IRF-1. The cells were exposed to IFN-β (200 U/ml) or IFN-γ (0.5 μg/ml) for 48 h. CASP3 Caspase 3. **d**, **e** Flow cytometry assay of apoptotic sub-G1 fraction. Cells were cotransfected with siRNAs (20 pM) and expression plasmids (2 μg) as indicated and exposed to 5-FU (25 μM), etoposide (50 μM), or H_2_O_2_ (50 μM) for 48 h. Data represent means ± SD of triplicate assays. *p < 0.05, **p < 0.01 (Student’s *t* test). **f**, **g** The IRF-1−XAF1 interplay in suppression of tumor cell growth. HCT116 sublines (sh-Control and sh-IRF-1) and DU145 sublines (sh-Control and sh-XAF1) were transfected with XAF1 and IRF-1, respectively and its effect on colony-forming ability of the cells was compared. Data represent means ± SD of triplicate assays

To investigate the functional interplay of IRF-1 and XAF1 in tumor suppression, we initially characterized their interrelationship in stress-induced apoptosis. Both mRNA and protein levels of IRF-1 and XAF1 were markedly increased in response to various cytotoxic stresses, including genotoxic agents (etoposide and 5-FU), γ-irradiation (IR), hypoxia, and cytokines (TNF-α and IFN-γ) (Fig. 1a and Supplementary Figure 1a). Under the same conditions, however, *XAF1* mRNA induction was blocked if IRF-1 expression was repressed by siRNA-mediated depletion while IRF-1 protein induction was blocked if XAF1 expression was depleted. Moreover, stress-induced cleavage of poly (ADP-ribose) polymerase (PARP) was profoundly reduced by depletion of either IRF-1 or XAF1, supporting the crucial role for their interplay in apoptotic stress response. Using short hairpin (sh) RNA-mediated stable knockdown and IFN treatment, we confirmed that apoptosis-promoting function of XAF1 and IRF-1 is debilitated in sh-IRF-1 and sh-XAF1 cells, respectively (Fig. 1b, c). Flow cytometric analysis also revealed that IRF-1 and XAF1 increase apoptotic sub-G1 fraction in cells exposed to 5-FU, etoposide, and H_2_O_2_ and this effect is abolished by depletion of XAF1 and IRF-1, respectively (Fig. 1d, e). Consistently, XAF1 and IRF-1 decreased the colony-forming ability of tumor cells in a highly IRF-1- and XAF1-dependent manner, respectively (Fig. 1f, g). The off-target effect of siRNAs was excluded by usage of three different si-IRF-1s and si-XAF1s (Supplementary Figure 1b and c). Effect of RNA/DNA transfection-evoked IFN signaling on IRF-1 and XAF1 expression was excluded by testing effect of siRNA molecules and shRNA plasmids (Supplementary Figure 1d−f). These results support that IRF-1 and XAF1 form a feed-forward loop to promote apoptosis under cytotoxic stress conditions.

### XAF1 stabilizes IRF-1 in response to apoptotic stresses

**Fig. 2:**
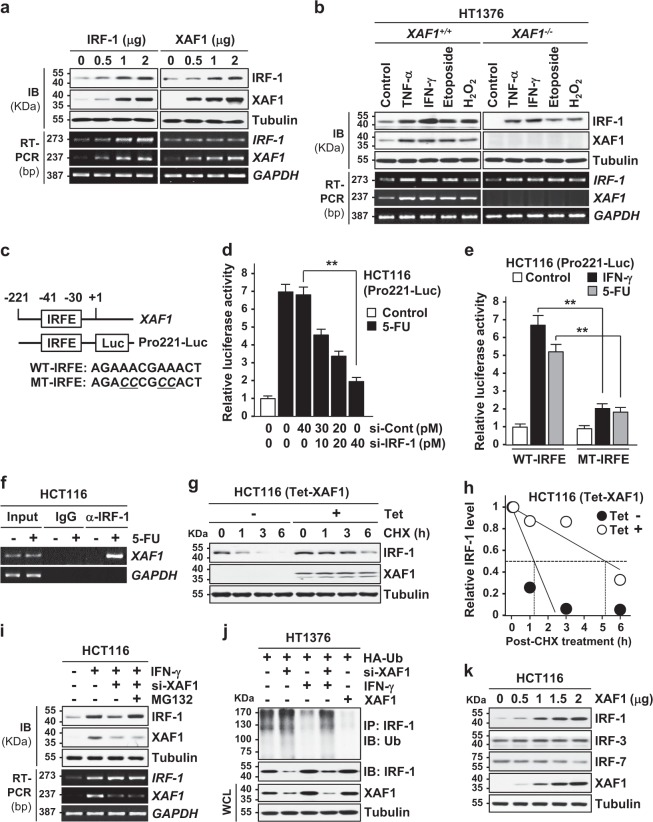
XAF1 is a feedback activator of IRF-1. **a** A mutual activation relationship of IRF-1 and XAF1. DU145 cells were transfected with increasing doses of IRF-1 or XAF1 and IB and RT-PCR assays were performed at 48 h after transfection. **b** Comparison of stress-mediated IRF-1 induction in HT1376 *XAF1*^*+/+*^ and its *XAF1*^*−/−*^ subline cells. Cells were exposed to TNF-α (50 ng/ml), IFN-γ (0.5 μg/ml), etoposide (50 μM), or H_2_O_2_ (50 μM) for 48 h. **c** A putative IRFE in the *XAF1* promoter and reporter construction for luciferase assay. **d**, **e** Attenuation of the Pro221-Luc responsiveness to 5-FU (25 μM, 12 h) and IFN-γ (0.5 μg/ml, 12 h) by IRF-1 depletion or IRFE mutation. Data represent means ± SD of triplicate assays. **p < 0.01 (Student’s *t* test). **f** ChIP assay showing IRF-1 binding to the IRFE within the *XAF1* promoter in 5-FU-treated cells. **g**, **h** CHX chase experiment showing XAF1 stabilization of IRF-1. Control and Tet (10 μg/ml, 12 h)-treated HCT116 (Tet-XAF1) cells were exposed to CHX (10 μg/ml) for indicated times and IB assay was performed to determine IRF-1 protein levels. **i** Effect of proteasome inhibition on XAF1 regulation of IRF-1. HCT116 cells transfected with si-Control or si-XAF1 were exposed to IFN-γ (0.5 μg/ml, 24 h). MG132 (10 μM) was added at 6 h before harvest. **j** XAF1 inhibition of IRF-1 ubiquitination. Ub ubiquitin, WCL whole cell lysate. **k** IB assay showing no detectable effect of XAF1 on protein levels of IRF-3 and IRF-7

Next we defined the molecular basis for the IRF-1−XAF1 interplay. As predicted, assays using ectopic transfection and comparison of stress-induced IRF-1 expression in HT1376 *XAF1*^*+/+*^ and *XAF1*^*−/−*^ sublines support that XAF1 upregulates IRF-1 protein expression while IRF-1 activates *XAF1* transcription (Fig. 2a, b). It was reported that *XAF1* transcription is activated by c-Jun N-terminal kinase (JNK1) through IRF-1^31^. Based on this, we tested whether IRF-1 directly activates *XAF1* transcription in stressed cells. A promoter luciferase assay using the XAF1-Pro221-Luc reporter, which comprises the IRF element (IRFE, nucleotides –30/–41 relative to ATG), revealed that the reporter responsiveness to 5-FU and IFN-γ is substantially decreased if IRF-1 is depleted or IRFE is mutated (Fig. 2c−e). Chromatin immunoprecipitation (ChIP) assay also revealed that IRF-1 binds to the IRFE within the *XAF1* promoter (Fig. 2f and Supplementary Figure 2a). Our previous studies demonstrated that XAF1 regulates protein stability through direct interaction with ubiquitin E3 ligases^9,32^. To address whether XAF1 enhances the protein stability of IRF-1, a cycloheximide (CHX) chase experiment was performed using tetracycline-inducible XAF1 system (HCT116/Tet-XAF1). XAF1 induction by tetracycline addition led to an increase in the half-life of IRF-1 protein from approximately 1.2 to 5.2 h while tetracycline itself did not affect IRF-1 level (Fig. 2g, h and Supplementary Figure 2b and c). Moreover, IRF-1 reduction caused by XAF1 depletion was blocked by the proteasome inhibitor MG132, and ubiquitinated IRF-1 level was up- and downregulated by XAF1 depletion and expression, respectively, supporting that XAF1 prevents the ubiquitination and proteasomal degradation of IRF-1 (Fig. 2i, j). Meanwhile, it was observed that XAF1 does not affect expression of other IRF family members, such as IRF-3 and IRF-7 (Fig. 2k). Collectively, these results indicate that under stressful conditions, IRF-1 is a key transcription factor for XAF1 activation and XAF1 plays a crucial role in IRF-1 stabilization and activation.

### XAF1 protects IRF-1 from CHIP-mediated ubiquitination through direct interaction

**Fig. 3:**
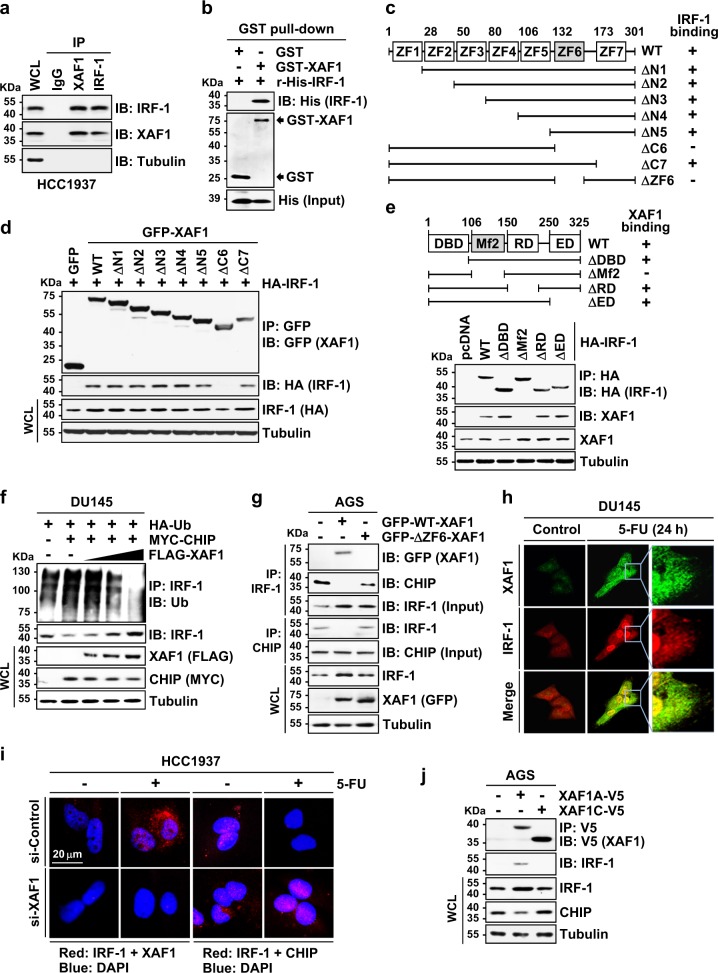
XAF1 binds directly to IRF-1. **a** An IP assay showing the interaction of XAF1 and IRF-1 expressed in HCC1937 cells. IP and IB for IRF-1 were done using rabbit and mouse antibody, respectively. **b** In vitro GST pull-down assay showing the direct interaction of purified GST-XAF1 and His-IRF-1 proteins. r Recombinant. **c** XAF1 constructs and its IRF-1-binding status. ZF zinc finger. **d**, **e** Identification of ZF6 and Mf2 as essential regions for the XAF1−IRF-1 interaction. DBD DNA binding domain, Mf2 multifunctional domain 2, RD regulatory domain, ED enhancer domain. **f** XAF1 inhibition of CHIP-mediated IRF-1 ubiquitination. **g** XAF1 competition with CHIP in binding IRF-1 and loss of IRF-1-binding activity of ΔZF6-XAF1. **h** Immunofluorescence microscopic analysis of XAF1 and IRF-1. Cells exposed to 5-FU (25 μM, 24 h) were incubated with anti-XAF1 or anti-IRF-1 antibody and proteins were visualized with secondary antibodies. **i** Proximity ligation assay for the IRF-1 interaction with XAF1 or CHIP. Cells transfected with siRNAs (20 pM) were exposed to 5-FU (25 μM, 24 h). Incubation with primary antibodies and PLA probes and amplification using polymerase were performed according to the manufacturer’s instruction. DAPI was used for counterstaining of the nuclei. **j** An IP assay showing no activity of XAF1C to bind and induce IRF-1

To dissect the molecular mechanism underlying XAF1-induced IRF-1 stabilization, we asked whether XAF1 binds to IRF-1. An immunoprecipitation (IP) assay for endogenous IRF-1 and XAF1 expressed in HCC1937 cells and in vitro pull-down assay using purified GST-XAF1 and recombinant IRF-1 revealed that XAF1 binds directly to IRF-1 (Fig. 3a, b and Supplementary Figure 3a). Using a series of deletion mutants, we identified that the ZF6 domain of XAF1 and the multifunctional domain 2 (Mf2) of IRF-1 are crucial for their interaction and XAF1-mediated IRF-1 stabilization (Fig. 3c−e). Given that CHIP is a U box-carrying ubiquitin E3 ligase that binds to the Mf2 domain of IRF-1, we assessed if XAF1 competes with CHIP in binding IRF-1, thereby interfering with CHIP-mediated IRF-1 ubiquitination^29^. As predicted, IRF-1 protein level was decreased by WT-CHIP but not affected by the U box-deleted CHIP (ΔUBox-CHIP), confirming that CHIP downregulates IRF-1 via its E3 ligase activity (Supplementary Figure 3b and c). CHIP-mediated IRF-1 ubiquitination was suppressed by WT-XAF1 in a dose-associated manner but not affected by ΔZF6-XAF1 (Fig. 3f and Supplementary Figure 3d). Moreover, the CHIP−IRF-1 interaction was inhibited by WT-XAF1 but not by ΔZF6-XAF1, and their dissociation triggered by stresses was attenuated if XAF1 induction was blocked, indicating that XAF1 competes with CHIP in interaction with IRF-1 (Fig. 3g and Supplementary Figure 3e). In addition, it was seen that XAF1 expression leads to CHIP reduction, suggesting that XAF1 may also activate IRF-1 through CHIP downregulation (Fig. 3f). An immunofluorescence (IF) assay showed that XAF1 increases and colocalizes with IRF-1 in stressed cells (Fig. 3h). Likewise, proximity ligation assay (PLA) revealed an increase in the XAF1−IRF-1 interaction and a decrease in the CHIP−IRF-1 interaction in cells exposed to 5-FU or IFN-γ and an inhibitory role for XAF1 in the CHIP−IRF-1 interaction in these stressful conditions (Fig. 3i and Supplementary Figure 3f). Given that the full-length transcript of *XAF1* (*XAF1A*) is preferentially lost and short isoforms are upregulated in human tumors, we examined the IRF-1-stabilizing activity of XAF1C, a representative tumor-overexpressing variant lacking the ZF6 domain^7,8^. Unlike XAF1A, XAF1C failed to bind to and stabilize IRF-1 and also showed no activity to downregulate CHIP, suggesting that the isoform switch of XAF1 may cause the disruption of the IRF-1−XAF1 loop in tumorigenic process (Fig. 3j).

### XAF1 functions as a transcriptional coactivator of IRF-1

**Fig. 4:**
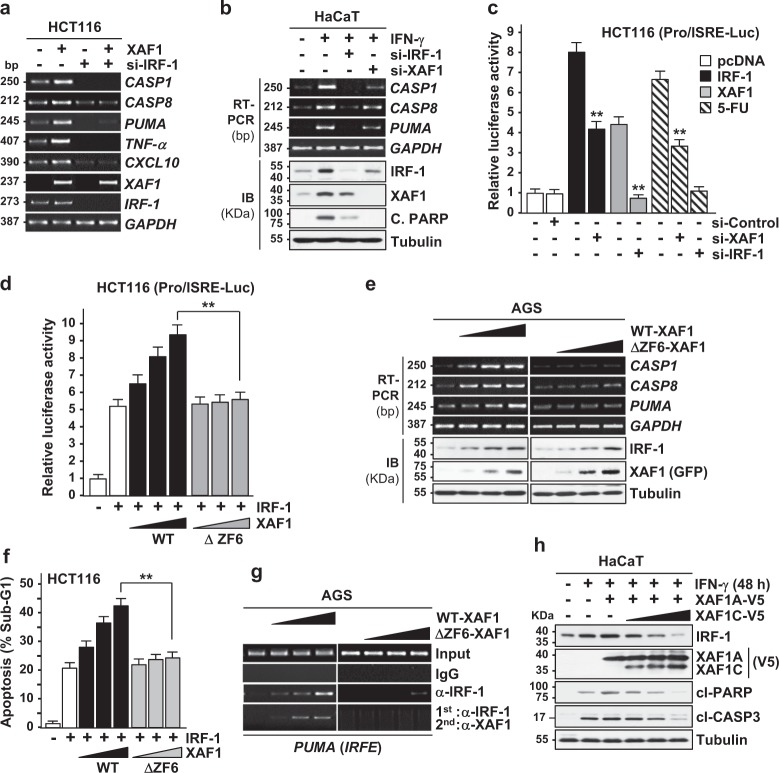
XAF1 functions as a transcriptional coactivator of IRF-1. **a** XAF1 activation of IRF-1 target genes. Cells were transfected with XAF1 and/or si-IRF-1 and after 48 h transfection, RT-PCR was performed to determine expression levels of IRF-1 target genes. **b** A role for XAF1 in IFN activation of IRF-1 target gene expression. Cells transfected with si-XAF1 or si-IRF-1 were exposed to IFN-γ (0.5 μg/ml) for 24 h. **c** XAF1 involvement in IRF-1 or 5-FU activation of the Pro/ISRE-Luc reporter. Cells were cotransfected with Pro/ISRE-Luc reporter and IRF-1 or XAF1. 5-FU (25 μM) was treated for 12 h. Data represent means ± SD of triplicate assays. **p < 0.01 (Student’s *t* test). **d**−**f** Comparison of WT- and ΔZF6-XAF1 in activation of the Pro/ISRE-Luc reporter, IRF-1 target genes, and apoptosis. Cells were transfected with the reporter, IRF-1, and increasing doses of either WT- or ΔZF6-XAF1 as indicated. Luciferase, expression, and flow cytometry analyses were carried out after 48 h transfection. **g** Sequential ChIP assay showing the complex formation of WT- but not of ΔZF6-XAF1 with IRF-1 on the IRFE region within the *PUMA* promoter. Cell lysates were precipitated with anti-IRF-1 antibody and the resulting complexes were precipitated with anti-XAF1 antibody. **h** Comparison of XAF1A and XAF1C effect on IFN-induced apoptosis. Cells transfected with XAF1A and XAF1C were exposed to IFN-γ (0.5 μg/ml) for 48 h

IRF-1 evokes growth inhibition effects mainly through its transactivation function^21–24^. We asked whether XAF1 stabilization of IRF-1 leads to an increase in the nuclear IRF-1 level and activates IRF-1-mediated transcription. XAF1 overexpression resulted in an increase in both the nuclear and the cytoplasmic IRF-1 and upregulated mRNA expression of IRF-1 target genes, such as *PUMA*, *CASP1, CASP8*, and *TNF-α* in an IRF-1-dependent manner (Fig. 4a and Supplementary Figure 4a). Likewise, in cells exposed to IFN-γ, blockade of XAF1 induction greatly attenuated IFN-γ activation of the IRF-1 targets, indicating XAF1 stimulates IRF-1-mediated transcription (Fig. 4b). A luciferase assay using the Pro/ISRE-Luc reporter harboring the IRFE showed that reporter activation by IRF-1 and XAF1 is significantly impeded by depletion of XAF1 and IRF-1, respectively (Fig. 4c). It was also shown that the reporter is activated by 5-FU and this is inhibited by depletion of XAF1 as well as IRF-1. As predicted, ΔZF6-XAF1 failed to activate the reporter, IRF-1 targets, and IRF-1-induced apoptosis (Fig. 4d−f). Furthermore, a sequential ChIP assay revealed that WT-XAF1 but not ΔZF6-XAF1 forms a complex with IRF-1 on the IRFE region within the *PUMA* promoter, supporting that XAF1 not only stabilizes IRF-1 but also activates its transactivation function through direct interaction (Fig. 4g and Supplementary Figure 4b). Consistently, XAF1C showed no activity to promote IFN-γ-induced apoptosis and rather impaired XAF1A’s proapoptotic effects, suggesting that XAF1C may act as a dominant-negative inhibitor against XAF1A (Fig. 4h). Together, these findings demonstrate that stress-induced XAF1 functions as an IRF-1 coactivator to stimulate apoptosis and suppress tumorigenesis.

### XAF1 suppresses NF-κB tumor-promoting function by reinforcing IRF-1 binding to coregulated target promoters

**Fig. 5:**
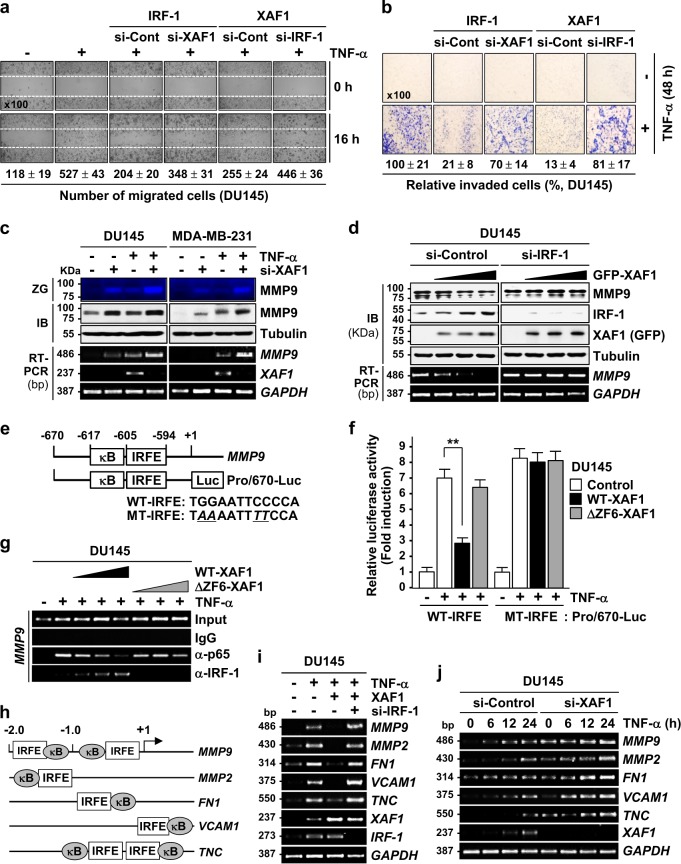
XAF1 represses p65/RelA-mediated *MMP9* transcription via IRF-1 activation. **a**, **b** Wound healing and Matrigel assays showing the suppression of tumor cell migration and invasion by the IRF-1−XAF1 interplay. DU145 cells were cotransfected with expression vectors and siRNAs as indicated and exposed to TNF-α (25 ng/ml). Using microscopic analysis, the number of migrated cells (wounded space) and invaded cells (blue color) was counted. Data represent means ± SD of triplicate assays. **c** Effect of XAF1 on TNF-α-mediated MMP9 induction. Cells transfected with either si-Control or si-XAF1 were exposed to TNF-α (20 ng/ml) for 20 h. ZG zymography. **d** XAF1 inhibition of MMP9 expression in an IRF-1-dependent manner. **e** Putative IRFE and κB site in the *MMP9* promoter and reporter construction for luciferase assay. **f** Comparison of WT- and ΔZF6-XAF1 effect on TNF-α activation of WT- and IRFE-mutated Pro/670-Luc reporter. Data represent means ± SD of triplicate assays. **p < 0.01 (Student’s *t* test). **g** Opposite effects of XAF1 on p65/RelA and IRF-1 interaction with the *MMP9* promoter. ChIP assay was performed using cells exposed to TNF-α (20 ng/ml) for 12 h. **h** Schematic representation for NF-κB target promoters harboring closely linked IRFE and κB site. FN1 fibronectin 1, VCAM1 vascular cell adhesion molecule 1, TNC tenascin C. **i**, **j** A repressive role for XAF1 in TNF-α activation of NF-κB target genes. Cells transfected with XAF1 and/or siRNAs were exposed to TNF-α (20 ng/ml). RT-PCR was performed to determine mRNA expression of NF-κB target genes

Matrix metalloproteinase 9 (MMP9) plays a key role in tumor cell migration and invasion induced by various tumor-promoting cytokines and growth factors, including TNF-α and EGF^33^. The IRFE and κB site are closely linked within the *MMP9* promoter and IRF-1 binding to the IRFE hinders p65/RelA binding to the κB site^34^. On this basis, we assessed whether XAF1 inhibits tumor cell migration and invasion by blocking p65/RelA-mediated *MMP9* transcription through IRF-1 induction. Wound healing and Matrigel assays revealed that IRF-1 and XAF1 strongly suppresses TNF-induced tumor cell migration and invasion and this effect is abrogated by depletion of XAF1 and IRF-1, respectively, indicating TNF-driven tumor malignancy is impaired by the IRF-1−XAF1 interplay (Fig. 5a, b and Supplementary Figure 5a, b). Both basal and TNF-induced MMP9 levels were up- and downregulated by XAF1 depletion and expression, respectively in the IRF-1-dependent manner (Fig. 5c, d). A luciferase assay showed that WT-XAF1 but not ΔZF6-XAF1 debilitates TNF activation of the MMP9-Pro/670-Luc reporter while this effect is not exerted for an IRFE-mutated reporter (Fig. 5e, f). Moreover, XAF1 stimulated IRF-1 binding to the IRFE within the *MMP9* promoter and this is accompanied with a decrease in p65/RelA binding to the κB site (Fig. 5g and Supplementary Figure 5c). Next we tested XAF1 effect on other NF-κB targets (*MMP2*, *FN1*, *VCAM1*, and *TNC*), which harbor the IRFE closed to κB site in their promoters (Fig. 5h). XAF1 inhibited TNF-α activation of these NF-κB targets in a highly IRF-1-dependent manner while XAF1 depletion caused further elevation of their expression (Fig. 5i, j). These support that XAF1 inhibits NF-κB tumor-promoting function by reinforcing IRF-1 interaction with a subset of coregulated target promoters.

### The IRF-1−XAF1 loop is commonly altered in human cancer

**Fig. 6:**
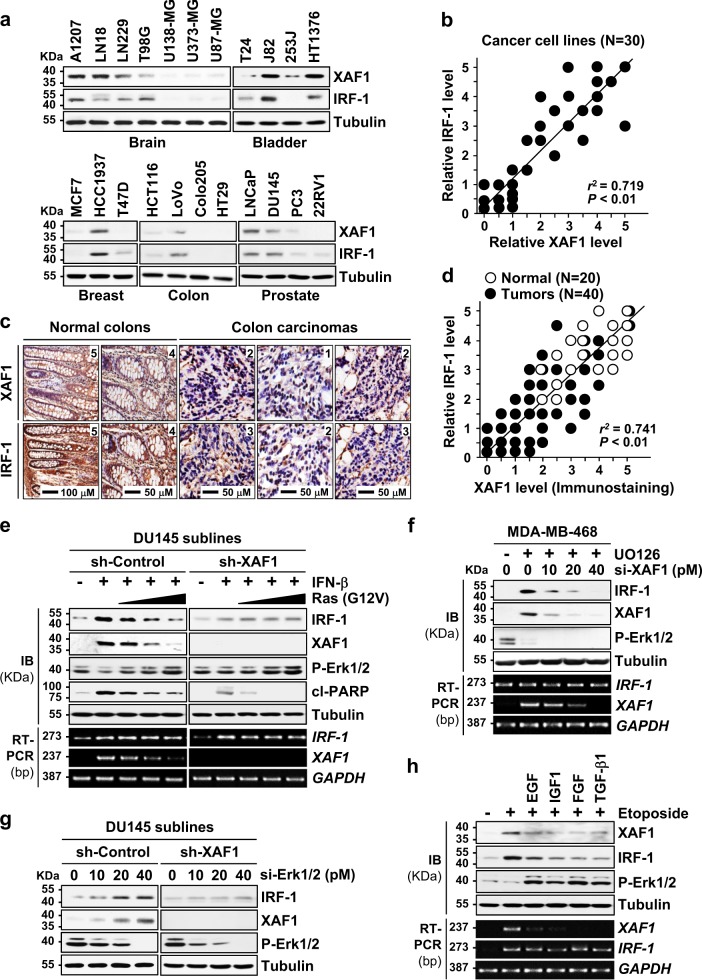
Frequent alteration of the IRF-1−XAF1 loop in human cancer. **a**, **b** A tight correlation of XAF1 and IRF-1 expression in human cancer cells. Relative expression levels were classified as levels 0−5. *r* Pearson’s correlation coefficient. **c**, **d** IHC study showing a strong correlation of XAF1 and IRF-1 immunoreactivity (dark brown) in human colon carcinoma and normal tissues. Purple staining indicates the nuclei. Relative staining levels were classified as levels 0–5. *r* Pearson’s correlation coefficient. **e** Ras suppression of IFN-mediated IRF-1 induction in a XAF1-dependent manner. Cells transfected with oncogenic H-Ras (G12V) were exposed to IFN-β (200 U/ml) for 48 h. **f** IRF-1 elevation by UO126 (10 μM, 8 h) and its blockade by XAF1 depletion. **g** IRF-1 induction by Erk1/2 depletion in an XAF1-dependent manner. IB assay was performed after 48 h si-Erk1/2 transfection. **h** Suppression of etoposide-mediated XAF1 and IRF-1 induction by Ras/MEK-activating growth factors. DU145 cells were incubated with EGF (50 ng/ml), IGF (100 ng/ml), FGF (50 ng/ml), or TGF-β1 (2 ng/ml) for 30 min and then exposed to etoposide (50 μM) for 16 h

To delineate the possible implication of the IRF-1−XAF1 axis alteration in tumorigenesis, we characterized expression status of IRF-1 and XAF1 in established cell lines and primary tumor tissues. Expression assay for XAF1 and IRF-1 in 30 human cancer cell lines of various origins and 60 primary colon carcinoma tissues revealed a strong correlation between XAF1 and IRF-1 levels (Fig. 6a−d). Immunohistochemical (IHC) study revealed that 18 (45%) and 20 (50%) of 40 primary tumors have ≤ 1.5 levels of XAF1 and IRF-1, respectively while all of 20 normal tissues tested show ≥ level 2.0 of XAF1 and IRF-1 (Fig. 6c, d). Moreover, 14 of 18 (77.8%) low XAF1 tumors and 14 of 20 (70%) low IRF-1 tumors displayed low IRF-1 and XAF1, respectively, indicating that XAF1 and IRF-1 levels correlate tightly in both normal and tumor tissues. It was reported that Ras/MEK activation increases tumor resistance to antiviral effects of IFNs by downregulating IRF-1 and that *XAF1* transcription is repressed by Ras/MEK^35–37^. We asked whether Ras/MEK downregulates IRF-1 through XAF1 repression. Oncogenic Ras (G12V) transfection abolished IFN induction of *XAF1* mRNA and IRF-1 protein but this effect was not seen in XAF1-depleted cells (Fig. 6e). Consistently, MDA-MB-468 cells with high EGFR level displayed a strong induction of *XAF1* mRNA and IRF-1 protein following treatment with the MEK inhibitor UO126 and this induction of IRF-1 was abolished if XAF1 is depleted (Fig. 6f). Likewise, Erk1/2 depletion led to IRF-1 induction in DU145/sh-Control cells but not in sh-XAF1 cells, supporting that Ras/MEK inhibits IRF-1 by repressing XAF1 expression (Fig. 6g). Additionally, we observed that Ras/MEK-activating growth factors suppress etoposide-mediated IRF-1 induction by blocking *XAF1* mRNA induction (Fig. 6h). Together, these support that the XAF1-IRF-1 axis is a target for oncogenic Ras-driven tumorigenesis.

### Disruption of the IRF-1−XAF1 axis contributes to tumor growth

**Fig. 7:**
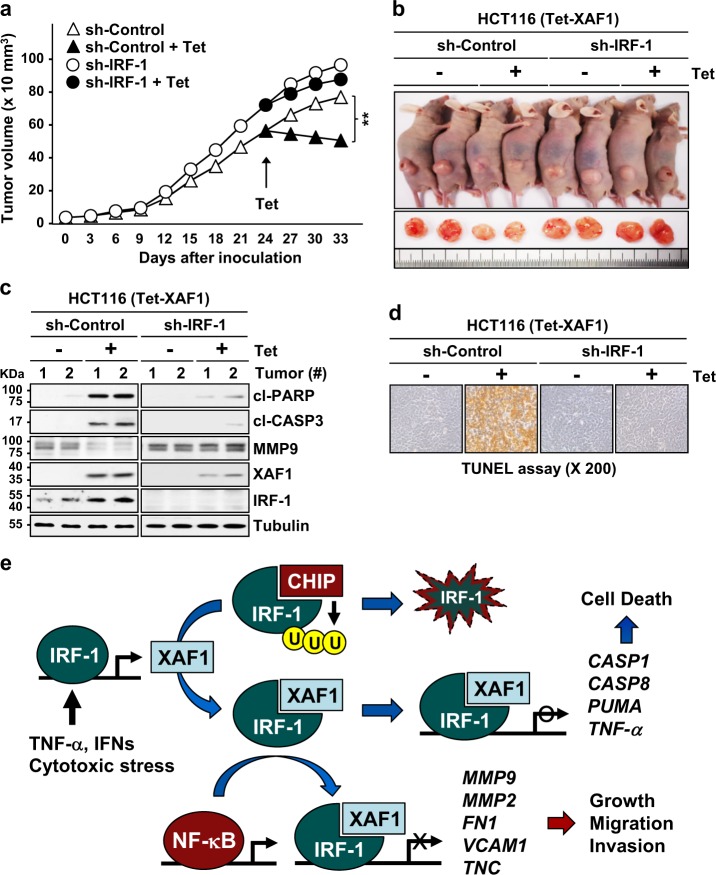
XAF1 suppresses tumor growth in an IRF-1-dependent manner. **a** Mouse tumor xenograft assay showing IRF-1 depletion effect on XAF1-mediated growth suppression. Data represent the mean ± SD (*N* = 6 per group; **p < 0.01). **b** Representative photographs of xenograft tumors at day 33 after inoculation. **c** Comparison of cleaved PARP and CASP3 and MMP9 levels in sh-Control and sh-IRF-1 tumors. **d** TUNEL assay of xenograft tumor tissues showing IRF-1 depletion effect on XAF1-induced apoptosis. Apoptotic nuclei (fragmented DNA) are stained dark brown. **e** Schematic representation of the IRF-1-XAF1 feedback loop and its role in cellular stress response

To elicit the role for the IRF-1−XAF1 interplay in vivo, we carried out mouse tumor xenograft assays using sh-Control and sh-IRF-1 sublines of HCT116 (Tet-XAF1) cells. As predicted, sh-IRF-1 tumors exhibited higher growth rate compared to sh-Control tumors (Fig. 7a, b). Following XAF1 induction by tetracycline injection, sh-Control tumors displayed a drastic regression whereas sh-IRF-1 tumors showed only mild response (37 versus 7%) (Fig. 7a). IB assay of tumor tissues revealed that XAF1 induction leads to MMP9 reduction and strongly increases both cleaved PARP and CASP3 levels in sh-Control but not sh-IRF-1 tumors (Fig. 7c). A TUNEL assay verified that XAF1-induced apoptosis is considerably attenuated in sh-IRF-1 versus sh-Control tumors (Fig. 7d). Collectively, our study identifies that XAF1 is a positive feedback regulator of IRF-1, which acts as a coactivator of IRF-1 to suppress tumorigenesis (Fig. 7e).

## Discussion

In the present study, we provide evidence that XAF1 binds directly to IRF-1 under stressful conditions and functions as a feedback activator of IRF-1 to induce apoptosis. Furthermore, XAF1 inhibits growth, migration, and invasion of tumor cells and suppresses in vivo tumor growth in a highly IRF-1-dependent manner, indicating that the tumor suppressive role of XAF1 is tightly linked to its ability to bind and activate IRF-1. We reported recently that XAF1 directs apoptotic switch of p53 function by modulating the p53-MDM2, Siah2-HIPK2, and ZNF313-p21^WAF1^ axes^9^. XAF1 was also shown to enhance cellular sensitivity to apoptotic stresses through the p53-independent mechanism and amplify TNF-α-induced apoptosis by activating the mitochondrial apoptotic pathway, supporting that XAF1 promotes apoptosis via multiple routes^4,10^. Our data demonstrate that XAF1 promotes apoptosis through the interplay with IRF-1 in multiple p53-deficient cancer cells, suggesting that the p53-independent function of XAF1 might be provoked through IRF-1 activation^18–20^. Together, this study adds a new XIAP- and p53-independent mechanism by which XAF1 acts as a proapoptotic tumor suppressor.

XAF1 controls protein stability through the regulation of ubiquitin E3 ligases, such as Siah2, ZNF313, cIAP2, and XIAP^9,38,39^. Consistent with this, we found that XAF1 is a novel IRF-1-stabilizing protein, which protects IRF-1 from CHIP-mediated ubiquitination. Our data identified two distinct mechanisms by which XAF1 stabilizes IRF-1. Firstly, XAF1 binds to IRF-1 and interferes with CHIP interaction with IRF-1. Secondly, XAF1 downregulates CHIP expression. IRF-1 stability is controlled mainly by the ubiquitin-proteasome system^29,30^. It was reported that CHIP is a major E3 ligase that limits growth inhibition activity of IRF-1 through the K48-linked polyubiquitination under certain stress conditions while it may play a positive role in the regulation of IRF-1 levels in unstressed cells^29^. Our study shows that XAF1 competes with CHIP in binding to the Mf2 region of IRF-1, thereby blocking the CHIP−IRF-1 interaction. The Mf2 region interacts with multiple IRF-1-destabilizing proteins, including CHIP, NPM1, TRIM28, and YB-1^18,29^. However, its role for the regulation of IRF-1 activity under stressful conditions remains to be characterized. In this context, our data support that Mf2 has a regulatory role in activation as well as stability of IRF-1, raising the possibility that the Mf2 region may serve as a sensor and/or switch that determines IRF-1 stress response by allowing the “docking” competition for multiple IRF-1 modulators with opposite functions. In this study, XAF1 was also found to downregulate CHIP protein level, supporting that XAF1 contributes to IRF-1 stabilization independently of its IRF-1-binding activity. Further studies will be required to define the molecular mechanism underlying the XAF1 regulation of CHIP. Nevertheless, our study demonstrates that through its IRF-1-binding property, XAF1 forms a complex with IRF-1 on the promoters of target genes, such as *PUMA*, *CASP8*, and *TNF-α*, to activate IRF-1-mediated transcription. This finding strongly suggests that XAF1 may reinforce IRF-1’s tumor suppression role by playing as a transcriptional coactivator of IRF-1 rather than simply enhancing its protein stability. It is also noticeable that IFNs activate IRF-1 at both mRNA and protein level. Our study demonstrates first that XAF1 plays a crucial role in IFN stabilization of IRF-1 by protecting IRF-1 from CHIP binding and subsequent ubiquitination-mediated proteasomal degradation. This observation supports that through the interplay with IRF-1, XAF1 might have an important role in IFN-mediated apoptosis and viral defense. Together, this study adds a new tumor suppression mechanism by which XAF1 functions as a coactivator of stress-inducible transcription factors, such as IRF-1.

NF-κB activates transcription of multiple genes involved in the regulation of cell survival or apoptosis in a cell type- and stimulus-dependent manner^40,41^. However, the mechanisms governing the target selectivity of NF-κB remain largely unknown. In the present study, we identified that XAF1 attenuates NF-κB-mediated transcription of tumor-promoting genes by facilitating IRF-1 repression of p65/RelA-mediated transcription. A previous study showed that IRF-1 function as a competitive inhibitor of p65/RelA in binding to the *MMP9* promoter, which comprises the IRFE overlapping or located closely to the κB site^34^. We observed that XAF1 stimulates IRF-1 binding to the IRFE, which is accompanied with reduced p65/RelA interaction with the κB site in multiple NF-κB target genes, including *MMP9*, *MMP2*, *VCAM1*, and *TNC*. These results suggest that XAF1 may switch the outcomes of NF-κB activation through the IRF-1-mediated repression of a specific subset of NF-κB target genes. Collectively, these demonstrate that XAF1 modulates IRF-1 function to stimulate and repress transcription of proapoptotic and tumor-promoting genes, respectively, thereby provoking its tumor suppressive function.

Multiple *XAF1* transcripts, including full-length (*XAF1A*) and short truncated (*XAF1B-E*), are expressed via alternative splicing in normal human tissues^5,7^. Interestingly, a switch from full-length to short transcripts was detected in tumors, raising the possibility that the truncated forms may have tumor-promoting functions or elicit a dominant-negative action against XAF1A^5,8^. Our recent study showed that XAF1C has substantially lower tumor suppression activity compared to XAF1A^9^. In the present study, we found that XAF1C, which lacks the IRF-1-binding domain, fails to activate IRF-1-mediated transcription and apoptosis. Moreover, XAF1C was shown to interfere with XAF1A’s apoptosis-promoting activity, lending support to the notion that XAF1C is a dominant-negative inhibitor of XAF1A and its preferential expression contributes to tumor progression. Given that both XAF1 and IRF-1 are frequently inactivated in multiple human cancers, it is conceivable that disruption of the XAF1−IRF-1 axis by loss of expression of XAF1 or IRF-1, isoform switch of *XAF1* transcript, or oncogenic activation of Ras/MEK signaling might be a common event that drives tumorigenesis. The restoration of a functional interplay of XAF1−IRF-1 could be an attractive avenue for the therapeutic intervention of tumor progression.

## Materials and methods

### Cell lines and reagents

Human cell lines (HCT116, DU145, HCC1937, HT1376, AGS, HaCaT, MDA-MB-231, and MDA-MB-468) were obtained from American Type Culture Collection (Rockville, MD, USA) or Korea Cell Line Bank (Seoul, Korea). All these cell lines were authenticated by short tandem repeat profiling, and allelic score data revealed a pattern related to the scores reported by the ATCC, and consistent with their presumptive identity. The HCT116 (Tet-XAF1) cells were generated by cotransfection of XAF1 (pcDNA4/TO) and tetracycline repressor vector (pcDNA6/TR) (Invitrogen, Carlsbad, CA, USA) as we described previously^9^. DU145 and HCT116 sublines with short hairpin (sh) RNA-mediated stable knockdown of XAF1 or IRF-1 were established by transfection of sh-XAF1, sh-IRF-1 or sh-Control constructs (Genolution Pharmaceuticals Inc, Seoul, Korea) and Zeocin (Invitrogen) selection. Recombinant human TNF-α was obtained from R&D Systems (R&D Systems Inc., MN, USA). Cyclohexamide was purchased from Sigma Aldrich (Saint Louis, MO, USA).

### Expression plasmids and siRNA

Expression vectors for XAF1, IRF-1, and CHIP were constructed using a PCR-based approach as previously described^5,9^. siRNA duplexes against *XAF1* (si-XAF1; 5ʹ-AUGUUGUCCAGACUCAGAG-3ʹ) and *IRF-1* (si-IRF-1; 5ʹ-CAGAUUAAUUCCAACCAA-3ʹ) were synthesized by Bioneer Inc (Daejeon, Korea). Control siRNA duplex served as a negative control was purchased from Dharmacon Research (Lafayette, CO, USA). Transfection of siRNAs or expression plasmids was performed using Neon^®^ Transfection System (Thermo Fisher Scientific, Waltham, MA, USA) or Turbofect™ in vitro Transfection Reagent (Pierce Biotechnology, Rockford, lL, USA).

### Chromatin immunoprecipitation (ChIP)

ChIP assay was carried out using a Simple ChIP™ Enzymatic Chromatin IP Kit (Cell Signaling Technology, Danvers, MA, USA) and antibodies specific for p65/RelA and IRF-1. PCR was done using primers 5F (sense; 5ʹ-TTACAACCTACAGTGTTCTA-3ʹ) and 6R (antisense; 5ʹ-AAGGGAAAGTGATGGAAGACT-3ʹ) for the *MMP9* promoter, PU-F (sense; 5′-GTAAGATCCATGTAAGTGATGTCAT-3′) and PU-R (antisense; 5′-AGACCCCATGCCAAATTTCATCCTG-3′) for the *PUMA* promoter, and CP8-F (sense; 5′-TATTTGCTACATAACTAAGAATGAA-3′) and CP8-R (antisense; 5′-CAAACATAGGTGTAAGTGCCCACTT-3′) for the *Caspase-8* promoter.

### Semi-quantitative RT-PCR analysis

Our strategy for the semi-quantitative RT-PCR analysis was described^3–5^. Briefly 1 μg of total cellular RNA was converted to cDNA by reverse transcription using random hexamer primers and MoMuLV reverse transcriptase (Invitrogen). PCR was initially performed over a range of cycles (20–40 cycles) by using serially diluted cDNA, and 1:4 diluted cDNA (12.5 ng per 50 μl of PCR) undergoing 24–38 cycles was found within the logarithmic phase of amplification with primers used for *XAF1* (sense; 5ʹ-CAGAAGTCCTCGCTGGAGTTTC-3ʹ and antisense; 5ʹ-TGAAATTCTTTCCCCTTTCC-3ʹ), *IRF-1* (sense; 5ʹ-ATGCCCATCACTCGGATGCGCAT-3ʹ and antisense; 5ʹ-GATATCTGGCAGGGAGTTACA-3ʹ), *MMP9* (sense; 5ʹ-ATACCTGTACCGCTATGGTT-3ʹ and antisense; 5ʹ-AACTCGTCATCGTCGAAATG-3ʹ), and an endogenous expression standard gene *GAPDH*. PCR was performed in 1.5 mM MgCl_2_-containing reaction buffer. Ten 10 μl of PCR products were resolved on 2% (wt/vol) agarose gels. Quantitation was achieved by densitometric scanning of the ethidium bromide-stained gels. Integration and analysis was performed by using Quantity One software program (Bio-Rad, Hercules, CA, USA).

### Immunoblot, immunoprecipitation, and immunohistochemistry

Immunoblot and immunoprecipitation assays were performed as described previously^9,32^.

Antibodies specific for XAF1 (SC-19194, ab17204), IRF-1 (SC-497, SC-514934), IRF-3 (SC-9082), IRF-7 (SC-9083), CHIP (SC-33264, SC-66830), cleaved PARP (CST #9541), cleaved CASP3 (CST #9661S), p65/RelA (SC-372, SC-514451), MMP-9 (CST #3852), phospho-Erk1/2 (CST #9101), U1 SnRNP 70 (SC-9571), anti-HA (SC-7392, SC-805, A2095), anti-GFP (SC-9996, ab69314), anti-Flag (SC-166384), anti-V5 (SC-83849, A7345), anti-Myc (SC-40), anti-His (SC-803), anti-GST(SC-33613), anti-Ubi (CST #8081), and β-tubulin (T0198) were purchased from Santa Cruz Biotechnology (Santa Cruz, CA, USA), Cell Signaling Technology, Abcam (Cambridge, MA, USA), or BD Bioscience (Franklin Lakes, NJ, USA). To discriminate IRF-1 from the similar size of Ig heavy chain, IP and IB for IRF-1 were carried out using rabbit and mouse antibodies, respectively. Immunohistochemistry assay for human colon tissues was performed using tissue arrays (US Biomax, Inc., Rockville, MD, USA) and Vectastain ABC (avidin-biotin-peroxidase) kit (Vector Laboratories, Burlingame, CA. USA) as described previously^32^. Briefly, slides were incubated with XAF1 or IRF-1 antibody overnight using biotin-free polymeric horseradish peroxidase-linked antibody conjugate system. Slides were counterstained with hematoxylin, dehydrated and visualized using an Olympus CK40 microscopy (Tokyo, Japan). For the immunoreactive score, we established a 1- to 12-point system by multiplying the percentage of positive cells by the intensity of the staining score. Two pathologists performed the assessment of immunostaining sections. Immunoreactive scores of 0–5 were classified as negative (level 0) and scores of 6–12 were regarded as positive (levels 1−5).

### Protein pull-down and in vitro binding assay

GST pull-down assays was performed as described^32,42^. For in vitro binding assay, GST-fused XAF1 (GST-XAF1) proteins overexpressed by IPTG in BL21 strain were purified using Glutathione Sepharose 4B (GE Healthcare, Little Chalfont, UK). N-terminal His-tagged recombinant human IRF-1 (r-His-IRF-1) proteins were purchased from Biomatik (Wilmington, DE, USA). The GST-XAF1 and r-His-IRF-1 proteins were incubated with binding assay buffer for 6 h at 4 °C. Immunocomplexes were separated by incubation with protein-A/G Sepharose for 1 h, heated at 95 °C in SDS sample buffer, and subjected to SDS/PAGE for immunoblot analysis.

### Immunofluorescence assay

Cells were fixed with 4% formaldehyde, permeabilized with 0.2% Triton X-100 in PBS, and blocked with 2% BSA and 0.1% Triton X-100 in PBS. Cells were incubated overnight with anti-XAF1 (ab17204, Abcam) or anti-IRF-1 (SC-514934) antibody at 4 °C, and stained with secondary antibodies. After mounting the coverslips, fluorescent imaging was obtained with a confocal laser scanning microscopy (LSM700, Carl Zeiss MicroImaging, Inc).

### Proximity ligation assay

Proximity ligation assay was performed using the Duolink^®^ In Situ Orange Starter Kit (DUO92102, Sigma) according to the manufacturer’s instruction. Briefly, after fixation and permeabilization, cells were incubated with primary antibodies (ab17204 for XAF1, SC-514934 for IRF-1, SC-66830 for CHIP) and then incubated with anti-rabbit PLUS and anti-mouse MINUS PLA probes for 1 h and ligation buffer for 30 min. For amplification step, the slides were incubated with amplification solution containing polymerase for 100 min at 37 °C. After DAPI staining, protein complexes in the cells were analyzed by confocal microscopy.

### Ubiquitination assay

Cells were incubated with MG132 (5 μM) for 4 h and cell extracts were prepared in buffer containing complete protease inhibitor (Roche) and deubiquitinase inhibitor *N*-Ethylmaleimide (E3876; Sigma Aldrich). The lysates were incubated with IRF-1 or anti-HA antibody overnight at 4 °C, and protein complexes were pelleted with protein A-agarose beads (Thermo Fisher Scientific, Waltham, MA, USA) and separated by SDS/PAGE. Ubiquitinated IRF-1 was immunoblotted with anti-HA or anti-IRF-1 antibody.

### Apoptosis and colony formation assay

Flow cytometric analysis of apoptotic sub-G1 fraction and Annexin V expression were performed using FACScan flow cytometer (Becton Dickinson) and CellQuest software (Becton Dickinson). For colony formation assay, 1×10^5^ cells per dish were maintained in the presence of G418 (1600 μg/ml) for 4−6 weeks. Selection medium was replaced every 2 days. Colonies were fixed with methanol for 15 min and stained with 0.05% crystal violet in 20% ethanol.

### Wound healing, cell tracking, and tumor invasion assay

For wound healing assay, cells grown to 100% confluency were scratched with a pipette tip and incubated in the absence and presence of TNF-α (25 ng/ml). Cell migration was monitored with a JuLi™ Br (Live cell movie analyzer, NanoEnTek Inc., Seoul, Korea). Invasion assay was performed using BD Matrigel™ matrix (BD Biosciences) and Polycarbonate Membrane Transwell^®^ Inserts (Corning, Corning, NY, USA). Cells were added to the matrigel-coated insert well and incubated for 48 h in the absence or presence of TNF-α (25 ng/ml). The invaded cells on the lower surface were stained with Diff-Quik staining kit (Sysmex, Kobe, Hyogo Prefecture, Japan). The number of stained cells per field was counted under a microscope at a magnification of ×200.

### Gelatin zymography for MMP9 activity

Cells were harvested with lysis buffer (25 mM Tris-HCl, pH 7.5, 100 mM NaCl, 1% IGEPAL CA-630, and protease inhibitors) and 40 μg of lysates were mixed with 5× sample buffer (without β-mercaptoethanol). Protein samples were run on the gel (10% polyacrylamide−0.1% gelatin gel) for 4 h. The gel was incubated in 1× renaturing buffer (2.5% Triton X-100) and then in 1× developing buffer (50 mM Tris-HCl pH 8.0, 0.2 M NaCl, 5 mM CaCl_2_, 0.02% NaN_3_) for 14 h. The gel was stained with Coomassie Blue R250.

### Terminal deoxynucleotidyl transferase-mediated deoxyuridine triphosphate nick-end labeling (TUNEL) assay

TUNEL assay for extracted xenograft tumors were performed using a DeadEnd™ Colorimetric TUNEL System Kit (Promega #G7360). Briefly, tumor cells were fixed with 4% paraformaldehyde in PBS, and incubated at 4 °C for 15 min with the buffer containing 3% bovine serum albumin and 0.1% Triton X-100. Signals were visualized directly under microscopy.

### Animal studies

Four-week-old immune-deficient male nude mice (nu/nu) (Orient Bio Inc., Sungnam, Korea) were maintained in pressurized ventilated cages. Tumor xenograft assay was carried out as described^32,42^. Briefly, the identical numbers (4×10^6^) of HCT116 (Tet-XAF1) sh-Control and sh-XAF1 subline cells were injected s.c. into six mice. Tumor growth was monitored periodically, and volume (*V*) was calculated by using the modified ellipsoidal formula: *V* = 1/2 × length × (width)^2^. At day 24, four mice of each group were exposed to saline or tetracycline by intratumoral injection, and tumor volume was measured at the beginning of injection and monitored regularly for 9 days. All studies were performed with the approval of Korea University Institutional Animal Care and Use Committee (IACUC) and Korea Animal Protection Law.

### Statistical analysis

Reporter luciferase, flow cytometry, cell viability, apoptosis, migration, and invasion assays were performed in triplicates, and data were presented as mean ± SD. Student’s *t* test was used to determine the statistical significance. A *p* value of <0.05 was considered significant.

## Electronic supplementary material

Legend for Supplementary Figures

Supplementary Figures 1-5
