# Supplementary material for: XAF1 forms a positive feedback loop with IRF-1 to drive apoptotic stress response and suppress tumorigenesis
Source: Cell Death Dis. 2018 Jul 24;9(8):806. doi: 10.1038/s41419-018-0867-4 (PMC6057933; doi:10.1038/s41419-018-0867-4)
Supplement: Supplementary file 1 — Legend for Supplementary Figures [file 41419_2018_867_MOESM1_ESM.docx]

**Legends for supplementary Figures**

**Fig. S1. a** The inter-relationship of IRF-1 and XAF1 activation in cells exposed to IFN-γ. HCT116 cells were transfected with siRNA (20 pM) as indicated and exposed to IFN-γ (0.5 μg/ml). Immunoblot (IB) and RT-PCR (reverse transcription-PCR) assays were performed to determine IRF-1 and XAF1 protein and mRNA expression levels after 48 h exposure. **b**, **c.** Knockdown efficiency of three different siRNAs against IRF-1 and XAF1. HCT116 cells were transfected with three different si-IRF-1s or si-XAF1s (#1-3, 20 pM) and treated with 5-FU (25 μM, 24 h). **d** No detectable effect of siRNA transfection on expression of IRF-1 and XAF1. HCT116 cells were transfected with an increasing dose of si-Control and IB and RT-PCR assays were performed to examine its effect on expression of IRF-1 and XAF1. **e, f** Comparison of IRF-1 and XAF1 expression levels in parental, sh-Control, sh-IRF-1, or sh-XAF1 cells.

**Fig. S2. a** An immunoblot of IRF-1 expression for ChIP assay (Fig. 2f). ChIP assay was performed to define IRF-1 binding to the IRFE within the *XAF1* promoter in 5-FU-treated cells. **b** CHX chase experiment showing the IRF-1 stabilization by Tet-induced XAF1. HCT116 (Tet-XAF1) cells transfected with either si-Control or si-XAF1 were exposed to Tet (10 μg/ml, 12 h). The cells were exposed to CHX (10 μg/ml) for indicated times and IB assay was performed to determine IRF-1 and XAF1 protein levels. **c** No detectable effect of Tet on IRF-1 protein level. Parental HCT116 cells were incubated with Tet (10 μg/ml, 12 h) and then exposed to CHX (10 μg/ml) for indicated times.

**Fig. S3. a** Test for antibody combination for the discrimination of IRF-1 from Ig heavy chain in IP assay. **b**, **c** Construction of WT and Ubox-deleted mutant CHIP and comparison of their IRF-1-downregulating activity. **d** Comparison of WT- and ΔZF6-XAF1 activity to inhibit CHIP-mediated IRF-1 ubiquitination. **e** IP assay showing activation of the XAF1-IRF-1 interaction and inactivation of the CHIP-IRF-1 interaction in HCC1937 cells after exposure to 5-FU (25 μM), H_2_O_2_ (50 μM), or IFN-γ (0.5 μg/ml) for 24 h. **f** Proximity ligation assay for the IRF-1 interaction with XAF1 or CHIP. Cells transfected with siRNAs (20 pM) were exposed to IFN-γ (0.5 μg/ml, 24 h). Incubation with primary antibodies and PLA probes and amplification using polymerase were performed according to the manufacturer’s instruction. DAPI was used for counterstaining of the nuclei.

**Fig. S4. a** Cell fractionation and IB assays showing XAF1 effect on the nuclear (N) and cytoplasmic (C) IRF-1. Cells were transfected with either si-Control or si-XAF1 and exposed to 5-FU (25 μM) for 12 h. U1 snRNP70 was used as a nuclear marker protein. **b** An immunoblot of IRF-1 and XAF1 expression for a sequential ChIP assay (Fig. 4g). Sequential ChIP assay was performed to define the complex formation of XAF1 with IRF-1 on the *PUMA* promoter. Cell lysates were precipitated with anti-IRF-1 antibody and the resulting complexes were precipitated with anti-XAF1 antibody.

**Fig. S5. a, b** Statistical analysis for wound healing and Matrigel assay data shown in Fig. 5a, b. Data represent means ± SD of triplicate assays. ** *p* < 0.01 (Student *t* test). **c** An immunoblot of p65 and IRF-1 expression for ChIP assay (Fig. 5g). A ChIP assay were performed to define the opposite effects of XAF1 on p65/RelA and IRF-1 interaction with the *MMP9* promoter. Cells were exposed to TNF-α (20 ng/ml, 12 h).
