# Supplementary material for: XAF1 forms a positive feedback loop with IRF-1 to drive apoptotic stress response and suppress tumorigenesis
Source: Cell Death Dis. 2018 Jul 24;9(8):806. doi: 10.1038/s41419-018-0867-4 (PMC6057933; doi:10.1038/s41419-018-0867-4)
Supplement: Supplementary file 2 — Supplementary Figures 1-5 [file 41419_2018_867_MOESM2_ESM.pptx]

## Slide 1
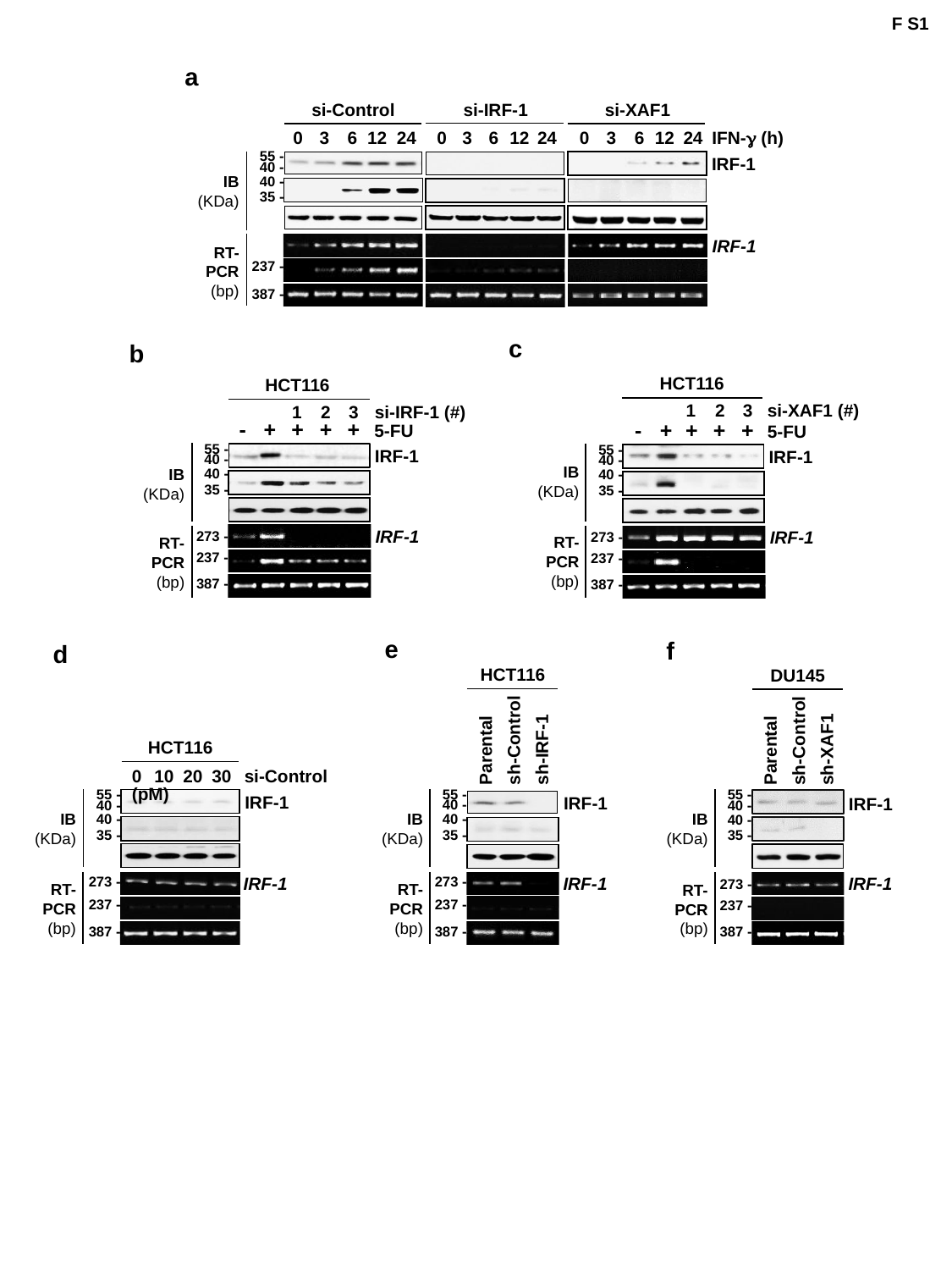

F S1
a
si-Control si-IRF-1 si-XAF1
0 3 6 12 24 0 3 6 12 24 0 3 6 12 24 IFN-γ (h)
55 -
40 -
40 -
35 -
55 -
273 -
237 -
387 -
IRF-1
XAF1
Tubulin
IB
(KDa)
RT-
PCR
(bp)
IRF-1
XAF1
GAPDH
c
b
HCT116
1 2 3 si-XAF1 (#)
- + + + + 5-FU
55 -
40 -
40 -
35 -
55 -
273 -
237 -
387 -
IRF-1
XAF1
Tubulin
IB
(KDa)
RT-
PCR
(bp)
IRF-1
XAF1
GAPDH
HCT116
1 2 3 si-IRF-1 (#)
- + + + + 5-FU
55 -
40 -
40 -
35 -
55 -
273 -
237 -
387 -
IRF-1
XAF1
Tubulin
IB
(KDa)
RT-
PCR
(bp)
IRF-1
XAF1
GAPDH
e
f
d
HCT116
Parental
sh-Control
sh-IRF-1
55 -
40 -
40 -
35 -
55 -
273 -
237 -
387 -
IRF-1
XAF1
Tubulin
IB
(KDa)
RT-
PCR
(bp)
IRF-1
XAF1
GAPDH
DU145
Parental
sh-Control
sh-XAF1
55 -
40 -
40 -
35 -
55 -
273 -
237 -
387 -
IRF-1
XAF1
Tubulin
IB
(KDa)
RT-
PCR
(bp)
IRF-1
XAF1
GAPDH
HCT116
0 10 20 30 si-Control (pM)
55 -
40 -
40 -
35 -
55 -
273 -
237 -
387 -
IRF-1
XAF1
Tubulin
IB
(KDa)
RT-
PCR
(bp)
IRF-1
XAF1
GAPDH

## Slide 2
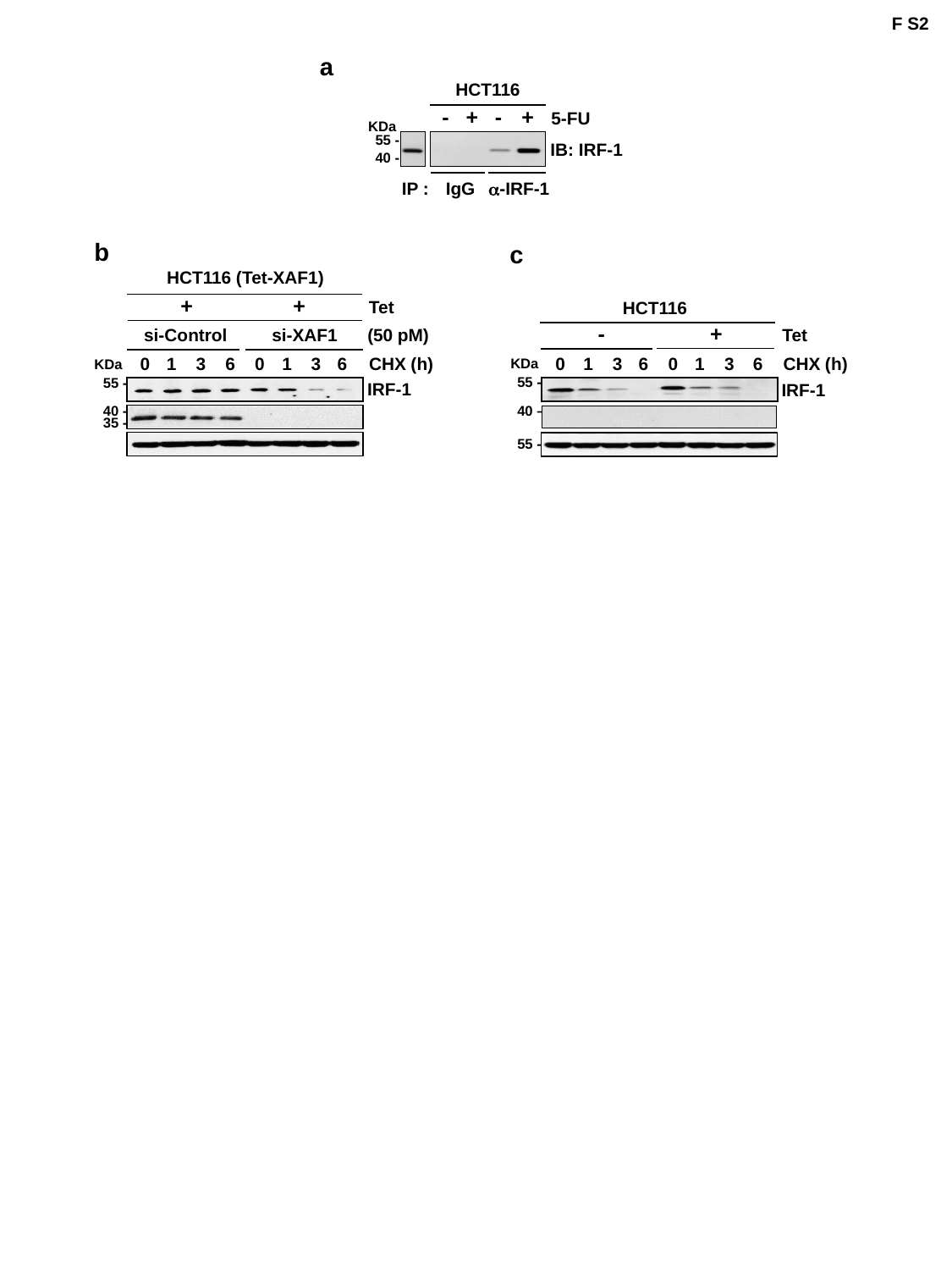

F S2
a
HCT116
- + - + 5-FU
KDa
55 -
40 -
IB: IRF-1
IP :
IgG α-IRF-1
b
c
HCT116 (Tet-XAF1)
+ + Tet
si-Control si-XAF1 (50 pM)
0 1 3 6 0 1 3 6 CHX (h)
KDa
55 -
40 -
40 -
35 -
55 -
IRF-1
XAF1
Tubulin
HCT116
- + Tet
KDa
0 1 3 6 0 1 3 6 CHX (h)
55 -
40 -
40 –
35 -
55 -
IRF-1
XAF1
Tubulin

## Slide 3
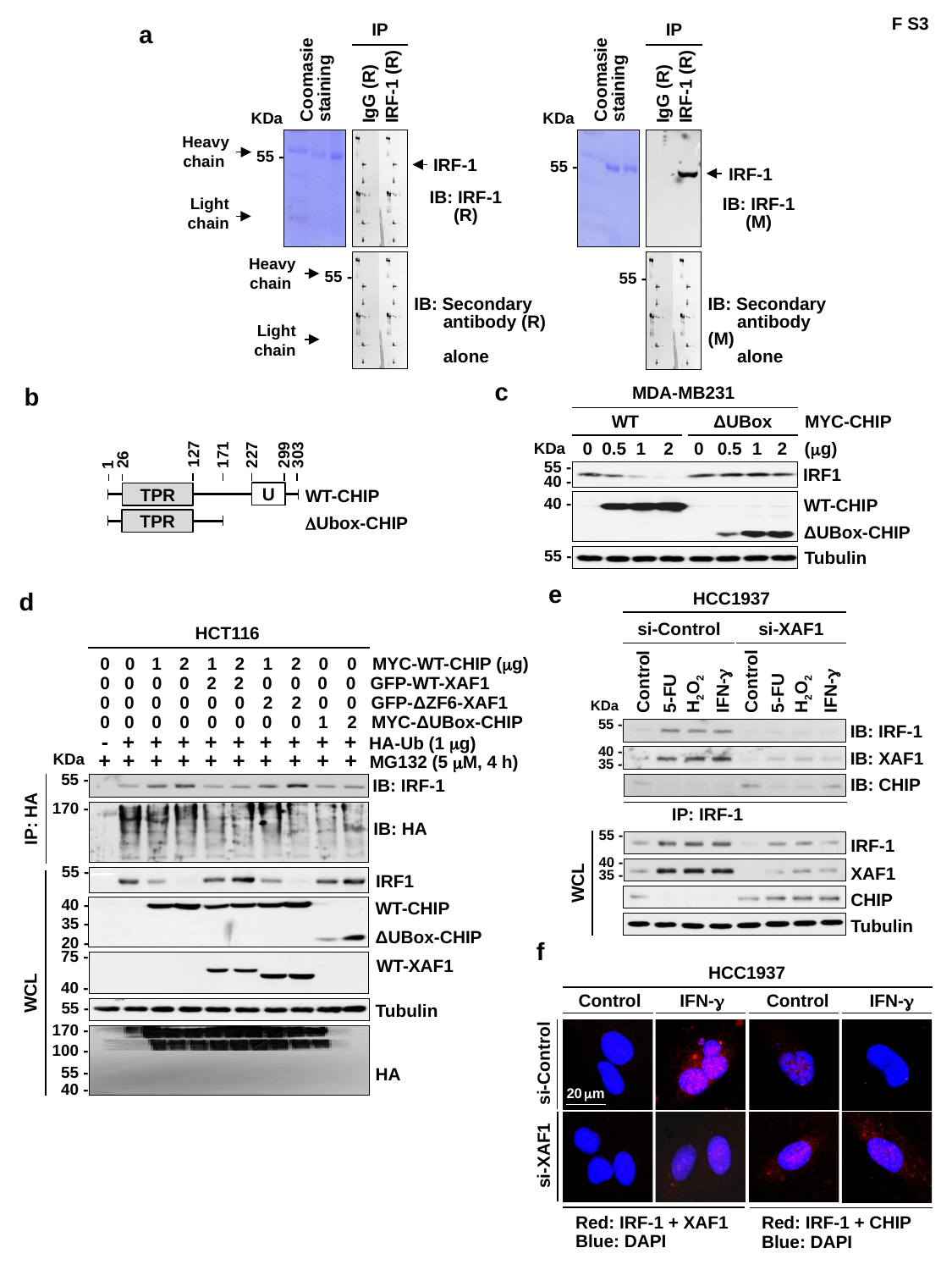

F S3
a
IP
IP
Coomasie
staining
Coomasie
staining
IgG (R)
IRF-1 (R)
IgG (R)
IRF-1 (R)
KDa
KDa
Heavy chain
Light chain
55 -
40 -
35 -
25 -
55 -
40 -
35 -
25 -
IRF-1
IRF-1
IB: IRF-1 (R)
IB: IRF-1 (M)
Heavy chain
Light chain
55 -
40 -
35 -
25 -
55 -
40 -
35 -
25 -
IB: Secondary
 antibody (R)
 alone
IB: Secondary
 antibody (M)
 alone
c
b
MDA-MB231
MYC-CHIP
WT
ΔUBox
0 0.5 1 2 0 0.5 1 2 (μg)
KDa
55 -
40 -
IRF1
40 -
35 -
20 -
WT-CHIP
ΔUBox-CHIP
55 -
Tubulin
 227
 299
 171
 127
 303
26
1
WT-CHIP
ΔUbox-CHIP
U
TPR
TPR
e
d
HCC1937
si-Control
si-XAF1
Control
5-FU H2O2
IFN-γ
Control
5-FU H2O2
IFN-γ
KDa
55 -
40 -
40 -
35 -
40 -
35 -
IB: IRF-1
IB: XAF1
IB: CHIP
IP: IRF-1
55 -
40 -
40 -
35 -
40 -
35 -
55 -
IRF-1
XAF1
CHIP
Tubulin
WCL
HCT116
0 0 1 2 1 2 1 2 0 0 MYC-WT-CHIP (μg)
0 0 0 0 2 2 0 0 0 0 GFP-WT-XAF1
0 0 0 0 0 0 2 2 0 0 GFP-ΔZF6-XAF1
0 0 0 0 0 0 0 0 1 2 MYC-ΔUBox-CHIP
- + + + + + + + + + HA-Ub (1 μg)
KDa
+ + + + + + + + + + MG132 (5 μM, 4 h)
55 -
40 -
IB: IRF-1
170 -
130 -
100 -
75 -
IP: HA
IB: HA
55 -
40 -
IRF1
40 -
35 -
20 -
WT-CHIP
ΔUBox-CHIP
75 -
55 -
40 -
WT-XAF1
ΔZF6-XAF1
WCL
55 -
Tubulin
HA
170 -
100 -
55 -
40 -
f
HCC1937
Control IFN-γ Control IFN-γ
20 μm
si-XAF1 si-Control
Red: IRF-1 + XAF1
Blue: DAPI
Red: IRF-1 + CHIP
Blue: DAPI

## Slide 4
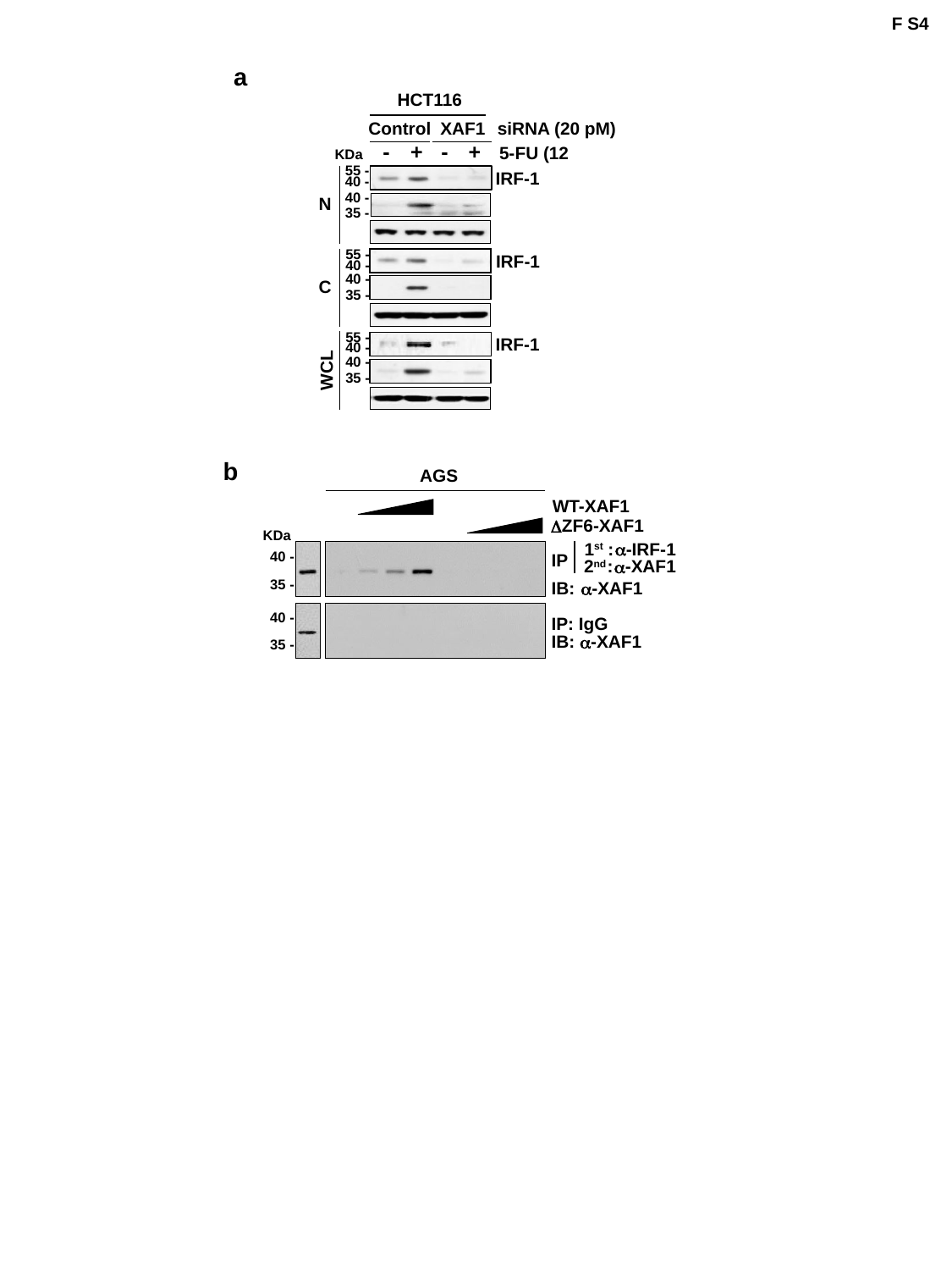

F S4
a
HCT116
Control XAF1 siRNA (20 pM)
KDa
- + - + 5-FU (12 h)
55 -
40 -
40 -
35 -
70 -
IRF-1
XAF1
U1 snRNP70
N
C
55 -
40 -
40 -
35 -
55 -
IRF-1
XAF1
Tubulin
55 -
40 -
40 -
35 -
55 -
IRF-1
XAF1
Tubulin
WCL
b
AGS
 WT-XAF1
ΔZF6-XAF1
KDa
1st : α-IRF-1
2nd : α-XAF1
40 -
35 -
IP
IB: α-XAF1
40 -
35 -
IP: IgG
IB: α-XAF1

## Slide 5
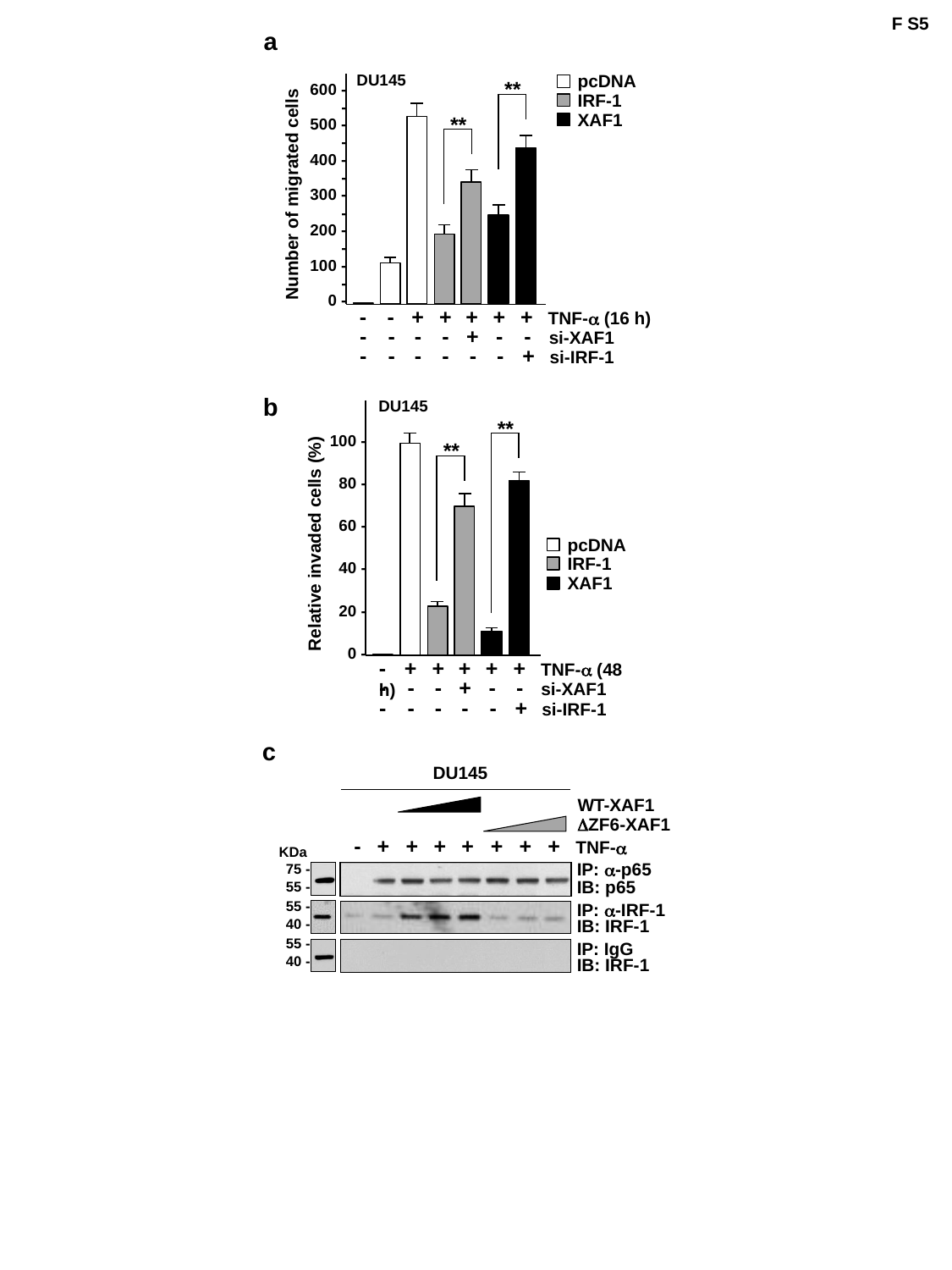

F S5
a
DU145
pcDNA
IRF-1
XAF1
**
600 -
500 -
400 -
300 -
200 -
100 -
0 -
 -
 -
 -
 -
 -
 -
**
Number of migrated cells
- - + + + + + TNF-α (16 h)
- - - - + - - si-XAF1
- - - - - - + si-IRF-1
b
DU145
**
**
100 -
80 -
60 -
40 -
20 -
0 -
Relative invaded cells (%)
pcDNA
IRF-1
XAF1
- + + + + + TNF-α (48 h)
- - - + - - si-XAF1
- - - - - + si-IRF-1
c
DU145
WT-XAF1
ΔZF6-XAF1
KDa
- + + + + + + + TNF-α
IP: α-p65
IB: p65
IP: α-IRF-1
IB: IRF-1
IP: IgG
IB: IRF-1
75 -
55 -
55 -
40 -
55 -
40 -
